# 

**Published:** 1892-04-02

**Authors:** 


### The Hospital

A WEEKLY INSTITUTIONAL JOURNAL OF

### Science, Medicine, Nursing, and Philanthropy.

159

EDITED BY

HENRY C. BURDETT,

AUTHOR OF "HOSPITALS AND ASYLUMS OF THE WORLD: THEIR ORIGIN, HISTORY, CONSTRUCTION, ADMINISTRATION, MANAGEMENT, AND LEGISLATION"; "HOSPITALS AND THE STATE"; "PAY HOSPITALS OF THE WORLD"; "COTTAGE HOSPITALS, GENERAL, FEVER, AND CONVALESCENT, WITH FIFTY BEDS AND UNDER": "BURDETT'S HOSPITAL ANNUAL AND YEARBOOK OF PHILANTHROPY"; AND "HELPS TO HEALTH."

ACTING EDITORS:

GEORGE W. POTTER, M.D.Edin.,

AUTHOR OF "MINISTERING WOMEN"; AND NUMEROUS PAPERS ON SCIENTIFIC AND MEDICAL SUBJECTS;

AND

P. WATSON WILLIAMS, M.D.Lond.,

PHYSICIAN TO THE OUT-PATIENTS AND FOR DISEASES OF THE THROAT, BRISTOL ROYAL INFIRMARY, ETC.

SURGEON GENL'S OFFICE
143512
LIBRARY

INDEX TO VOL. XII.

(2ND APRIL TO 24TH SEPTEMBER, 1892)

LONDON:
PUBLISHED BY THE SCIENTIFIC PRESS (LIMITED), 140, STRAND, W.C
1892.

W1 H069X

### INDEX TO VOL. XII. 1892.

Aberdeen Royal Infirmary, 408
Acute Pleuritis, Treatment of, 395
Addenbrooke's Hospital, Cambridge, 269
Adonis Vernalis, 299
After Thoughts, 199
Air and Water, 147
Alexandra Hospital Snowball, 424
Alice Memorial Hospital, Hong Kong, 32
Amœbic Dysentery, 74
Ambulance Conveyance, 136
Anæsthetics, 153
An Absurd Mistake, 422
Anarchism in Physic, 83
Anatomy, 204
An Extraordinary Lancashire Man, 135
An Imperial Medical Home and Club, 375
Apples, Presence of Arsenic in, 54
Aristotle's Method of Study, 423
Arnott's, Sir J., Gift, 360
Asaprol, 363
Asylum Administration, a New Departure, 109
——— or Hospital, 337
——— Attendant of To-day, The, 351
Asylums of the World:—I. History and Early Treatment, 223; II. Treatment, 255; III. Administration and Nursing, 270; IV. the Construction of Asylums, 301
Ayr County Hospital, 89

Bacteriology and the Medical Art, 321
Begging Children, 374
Belfast Hospital for Skin Diseases, 313
——— Royal Hospital, 377
Below Par, 295
Bischoff, Professor, on Brains, 406
Blood Poisoning, Curious Case of, 54
Board School Teachers, 311
Bolingbroke Pay Hospital, 88
Bradford Infirmary, 141
Brain Blood in Men and Women, 102
Bridgenorth and S. Shropshire Infirmary, 73
Brine Bath Treatment of Cholera, The, 390
British Home for Incurables, 280
——— Medical Association, The, and Women, 422
——— Medical Association, The, 280, 305
Brompton Consumption Hospital, 104, 345
Bryonia, 155
Bugs, 278

Calcium Salts, the Choice of, 348
Cancer as an Infective Disease, 7
Cancer Cure, 135
Carbolic Acid and its Preparation, 316
Cardiac Outlines, 412
Carmarthen County Infirmary, 249
Carnine Syrup, 188
Causation and Pathology of Rickets, The, 106
Centenary Collapse, A, 194
Central Council, A, 178, 206
Central London Throat and Ear Hospital, 185
——— Ophthalmic, 185
Charing Cross Hospital, 265
Cheese a Digestive, 168
Chesterfield Hospital, 281
Children's Hospital, Paddington, 137
——— Doctor and Farmer, 422
Cholera Precautions, 403, 424
——— Innoculation, 401
Chronic Pharingitis, 410
Chronic Indigence of Our Hospitals:
I., A Bird's-eye View, 13
II., Difficulties of Hospital Finance, 29
III., The Material Deficit, 45
IV., What Might Be, 61
Chronic Ulcers of the Legs, 90
Cigarette Smoking, 7
City of Dublin Hospital, 73
City of London Chest Hospital, 24, 104
City of London Truss Society, 88
Clergy and Vivisection, The, 71
Close Times, 114
Cold Ash Children's Hospital, 200
Colles's Fracture, 139
Colour Blindness, 352
Compulsory Vaccination, 198
Consumptives, Health Resort for, 38
———, A Paradise for, 247
Convalescent Home, Coatham, 105
——— for Children, Birmingham, 41
——— Bonchurch, 302
——— for Women, Proposed, 56
Cork County Hospital, 365
———, South, Infirmary and Female Students, 424
Coronilla varia, 315
Coryza, Abortive, The Treatment of, 330
Cost of Out-Patients, 380
Cremation and Poison, 326
Crisis of the Voluntary Hospital, The, 289
"Cures" for Habitual Drunkenness, 359, 431
——— of Poverty, The, 367, 383, 399, 415
Curiosities of Medical Politics, 119

Cystic Goiture, On Treatment of, 250

Damp Towels, 423
Deaths from Starvation, 40
Decrease of Population in France, 124
Dentistry for Poor Children, 136
Derby Hospital for Sick Children, 367
Derbyshire Hospital for Sick Women, 281
Diabetes, 411
Diet in Enteric Fever, 394
Disease the Monitor, 373
Disinfectants, 316
Dissection Guides, 91
Drainage of Ventricles in Meningitis, 154
Dress, Æsthetic, 22
Drunkenness and Crime, 28
Drunk or Dying, 25
Dumb Speak, How the, 23
Dust Bins and Cholera, 263

Edinburgh Dispensaries Closed, 376
Edinburgh Royal Infirmary, 56
Election Fever, 279
Elongated Uvula, 122
Employment for Women, 260
Empyena and Abscess of the Lung, 282
Ephedra Vulgaris, 396
Epilepsy and Paralysis, Hospital for, 8
Epiphora, 44
Erysipelas, 314
Evelina Hospital, 137

Facts about Cholera, 371
Faith Healing, 294
——— and Science at the Hospitals, 162
Fashion, 134
Festival Dinners:—King's College Hospital, 127; The Orphan Working School, 128; Clarence Memorial Wing, St. Mary's Hospital, 141; The Royal London Ophthalmic Hospital, 142; City of London Hospital for Diseases of the Chest, 159; North London Hospital for Consumption, 160; National Hospital for the Paralysed and Epileptic, 160
Fever Hospital Full, The, 391
——— Hospital, Proposed, 56
——— Hospitals and the *Chronicle*, 408
Finance, Hospital, 320
Fingers, Injuries to the, 43
First Lines of Defence, 369
——— Year's Men, To, 418
Fleming Memorial Hospital for Sick Children, Newcastle-on-Tyne, 430
Fossil Institution, A, 295
Friends in Need, 386
Funeral Reform, 38

Gangrene, 218
Gas, The Prometheus of, 327
Generous Nurse, A, 407
Gladstone, Mr., and the Medical Profession, 273
Glasgow Royal Infirmary and Female Students, 424
Gloucester Asylum, 31
Gordon Boys' Home, 296
Goschen, Mr., and Doctors' Incomes, 71
Gravesend Hospital, 185
Great Northern Central Hospital, 40, 152
Great Ormond Street Children's Hospital,
Great Writers, The Attitude of Towards Disease: I. Sir W. Scott, 52; II. Shakespeare, 116; III. French Satirists and Doctors, 148; IV. Jane Austen, 212; V. Lord Tennyson, 261; VI. Robert Burton, 277; VII. Dante, 293; VIII. Dickens, 310; IX. Dickens, His Medical Science, 324; X. Keats and Shelley, 340; XI. The Literature of Despair, 357

Hake's, Mr. Egmont, Book, 151
Hampstead Home Hospital, 24
Harvard Medical School, The, 268
——— University and Athletics, 406
Health and Beauty, 295
——— of Halifax, The, 199
Herts Convalescent Home, 376
Hills or Sea, 359
History and Capabilities of Herbal Simples, The Fig, 180; The Common Flag, 336; Garlick and Onions, 372
Hitchcock Memorial Hospital, Hanover, U.S.A., 15
Home Life for Working Girls, 151
——— Reading, 199
Homes for Little Boys, Farningham and Swanley, 136
Hop-Pickers' Protection Association, 406
Hors-de-Combat, 408
Horse Civilisation in Shetland and Wales, 87, 144
Hospital Administration, 92.
——— Administration—I. Organisation, 221; II. Admission of Patients, 253; III. Finance, 286. I. General Hospitals, 365; II. Special Hospitals, 381

Hospitals Association, 208
Hospital Boom, The, 70
Hospitals Closed for Repairs, 370
Hospital Committees, Trained Nurses and the Public, 257
——— Conference, The, 263
——— Construction, 349; Hall for Operations, Copenhagen, 32; Insane Asylum, Waterbury, Vermont, 47; A Hospital in Hong Kong, 48; Royal South London Ophthalmic Hospital, 78; Temporary Hospitals, 141; Radcliff Infirmary, Oxford, 141; Barraque Wards, 255; The New Hospital, Aden, 287; The Ladywell Sanatorium, Salford, 349; Forres Leanchoil Hospital, 398
——— for Epilepsy and Paralysis, 313
——— Parade, 376
——— Problem, 33
——— Saturday at Birmingham, 152
——— Saturday Fund, 342
——— for Sick Children, 24
——— for Women and Children, 281
Hospitals, The Work of:—I. Hospital Accommodation for Breadwinners, 169; II. Hospital Accommodation for Women, 171; III. What the Hospitals do for the Middle Classes, 172; IV. With the Children, 173; V. The Chronic Diseases of Children, Incurable Homes, and Other Supplemental Work, 175
———, The, and Hospital Sunday, 81
How not to Dine, 327
House Surgeon Question, The, 23
——— Governor or Medical Superintendent—I., 322; II., 338; III., 354
Hull, Royal Infirmary, Gift to, 360
Hygiene and Sanitary Science, 287
Hypnotism and Hysteria, 355
Hypermetropic Headaches, 999
Hyperæmia of the Conjunctiva, 107

Ice Cream and Enteric Fever, 292
Ideals of Science, The, 242
Impatient Science, 35
Impotent Oxford and its Melancholy Infirmary, 97
Inaudi Jacques, 264
Inebriates, 82
Inebriety, Society for the Study of, 272
Infanticide in China,
Infants, On the Feeding of, 58
Infectious Diseases in Private Houses, 176
Ingrowing Toe Nail, and Some Modern Instruments, 140
In Reversion, 66
Insane, Appeal of The, 6
——— Science, 99
——— The Story of the, from Year to Year, 6, 20, 86, 100, 150, 192, 396
Insanity, Outlines of, 267
Insanitary Cottages and the Authorities, 408
Insurance of Women, 280
Internal Hœmorrhage, 411
International Congress of Hygiene, 1894, 72
Interstitial Theratitis, 267
Irish Consumptive Hospital, the Proposed
Is Cholera Upon Us, 359
Isolation Block, Sunderland Infirmary, 344
Italian Hospital, 296
Italian Hospital, 297
Invalid Children's Aid Association, 318

Jewish and Gentile Children, 375
Joints, Affections of, 203
Juvenile Smokers, 98

Kidney, Symptoms of Surgical Affections, 12

Ladies' Chins, 71
Ladywell Sanatorium, Salford, 200
Laminectomy and Traumatic Compression, 138
Laryngeal Tuberculosis, Inhalation in, 11
Laundry, The, 397, 413
Leeds General Infirmary, 185
Leysin Sanitorium, 424
Lifeboat Saturday, A, 279, 300, 320
Liverpool Royal Infirmary, 137
Local Government Board and Fever Hospitals, 424
London Hospitals, Proposed Central Board, 302
London Temperance Hospital, 152
Longmore Hospital, 201
Lords, The, and the Nurses, 242
Lourdes Miracles, The, 407
Louth Dispensary and Infirmary, 249
Low Fever, Galen's Diagnosis of, 39
Lunacy in Scotland, 276
Lunacy Law, 188
Luther and Socialism, 406
Luton Bute Hospital, 264

Madmen and their Friends, 356
Male and Female Brains, 103

Manchester Crematorium, 364
—— Hospital for Consumption, &c., 72
—— Royal Infirmary, 344, 360
Manual of Chemistry, A, 204
Mary Wardell Convalescent Home, 265
Materia Medica, 252
Matteism, The Collapse of, 339
Meath Home for Epileptics, 344
Medical Aid to Children, 216
—— Annual, The, 60
—— Certificate, Tampering with 24
—— Examiner for Life Assurance, 1., 186; II., 202; III., 218
Medical History from the Earliest Times, II., Medicine in Prehistoric Times, 5; III., Medicine as Practised by Uncivilised Man, 21; IV., Medicine in Ancient Egypt, 37; V., Medicine in Ancient Egypt, 53; VI., Hindoo Medicine, 69; VII., Chaldean and Persian Medicine, 85; VIII, The Earliest Greek Medicine—Homer—Æsculapius, 101; IX., The Philosophers—Democedes—The Æsclepidæ, 117; X., Hippocrates, 133; XI., The School of Cos and Cnidus, 149; XII., The Successors of Hippocrates—The Dogmatic School, 165; XIII., The Alexandrine Anatomists, 181; XIV., Heraclides and the Empiric School, 195; XV., Military Medicine in Ancient Greece, 213; XVI., The Medical Profession in Ancient Greece, 245; XVII., Early Roman Medicine, 259; XVIII., The Methodic School, 275; XIX., Celsus and Ancient Surgery, 291; XX., Galen, 307; XXI., The Medical Profession in Rome, 325; XXII., Official Medicine in Ancient Rome, 341; XXIII., Roman Military Medicine 389; XXIV., The Influence of Christianity on Medicine, 405
Medical Schools and Hospitals, List of, 420
—— and Degrees, 419
Medical Sickness and Annuity, 343, 391
—— Year Book, A, 124
Medicine of the Future, 417
Mercury, Abuse of, in Eye Disease, 27
Metric Weights and Measures, 168
Metropolitan Drinking Fountains Association, 72, 280
—— Hospital Dinner, The, 272
—— Hospital Sunday Fund, 317
—— and Provincial Medical Schools, 426
Middlesex Hospital, 153
—— Hospital, 377
Military (Alexander) Hospital, Warsaw, The, 382
Milk and Scarlet Fever, 391
Mirth and Medicine, 167
Monkwearmouth Hospital, 344
More Room or Better Room, 17
Mortality of Foundlings, 118
Mouth Fauces, and Larynx, 364
Mucocele, 60
Mud and Muddle, 18
Multiplication of Metropolitan Hospitals, The, 228, 335
Must, 162
Mutual Help Brotherhood, 408

Nasal Polypi, On the Treatment of, 58
National Hospital for Paralysed and Epileptic, 216
New Beach Convalescent Home, Sandgate, 216
Newcastle Infirmary, 344
New Form of Hospital Sink, A, 111
—— Hospital for Women, 89
—— Time Coming, The, 55
—— York Children's Aid Society, 64
Nixon, Mr., Resignation, 431, 414
Noble's Isle of Man General Hospital, 328
Nocturnal Incontinence, Treatment of, 90
Northallerton Cottage Hospital, 89
North-Eastern Hospital for Children, 249
North London Hospital for Consumption, 104, 297
North-West London Hospital, 56, 201, 264
Not Out of Danger, 385
Nottingham General Hospital, 338
Nurses and Long Skirts, 215

Œsophagus, Diagnosis in the Structure of the, 187
Ophthalmic Hospitals, On the Special Requirements of, 109

Oxygen at Oxford, 3

Paddington Infirmary, 229
Paracentesis Pericardi, 298
Past and Present, 387
Pathology, Aids to General, 91
Payn's, Mr. James, Physicians, 247
Pembrokeshire and Haverfordwest Infirmary, 265
Pensions without Penalties, 50
Perityphlitis and Appendicitis, 347
—— The Question of Surgical Interferences, 74
Phagocytosis 1
Phenocoll Hydrochlorate, 284, 363
Pleurisy, The Present Treatment of Simple, 330
Pneumonia, The Death Rate of, 250
Poison and Poisoning:—Strychnia: Dr. Palmer, 68; The Marquise de Brinvilliers, 164
Poisoning, Phosphorus, Remedy for, 38
Poor Man's Gout, 131
Poplar Hospital for Accidents, 136
Popular Educator, A, 311
Post-Mortem Table, A New, 63
—— -Pharyngeal Abscess, 105
Practitioner's Bookshelf, 44
Premium on Inefficiency, A, 151
Prescriptions, Proprietorship of, 38, 86
Prison, A Model, 4
Prisons at Home and Abroad, 132
Public Health, 108
Pulse, The, 108
Pure Water, 406
Putting into Practice, 274

Quackery and Christianity, 380
Quacks, 374
Queen's Hospital, 153
Quinine and Malaria,

Radcliffe Infirmary, Oxford, 88, 95, 141
"Records," 327
Relations Between Chorea and Rheumatism, 378
Renal Calculus, Conditions Simulating, 282, 298
Report of the Lords Committee, The, 210
—— on Metropolitan Hospitals, The, 225
Resection of Intestines, 427
Rickets, Pathology of, 106
Royal Abert Edward Infirmary, Wigan, 376
—— Berks Hospital, Reading, 76
—— Consumptive, A, 263
—— Free Hospital, 73
—— Hants County Hospital, Winchester, 254
—— Hospital for Incurables, Putney, 302, 319
——, 342
—— Hospital for Women, 217
—— Infirmary and Medical School, Newcastle-on-Tyne, 429
—— London Ophthalmic Hospital, 142, 153
—— Medical Benevolent College, 72
—— National Hospital Consumption, Ventnor, 125
—— Portsmouth, Portsea, and Gosport Hospital, 205
—— Sea Bathing Infirmary, Margate, 108, 345
—— South Hants Infirmary Repairs, 392, 285
—— United Hospital, Bath, 360
—— Westminster Ophthalmic Hospital, 104
Rotherhithe Infirmary, Alterations at, 127
Rumination in Human Beings, 26

Samaritan Free Hospital for Women, 108
Sailors' Rests, 280
Salts of Strontium, 219
Scarlet Fever, 391
——, Treatment and Disinfection, 167
Salicylic Acid in Treatment of Rheumatism, 122
Salisbury Infirmary, 425
Salts of Strontium, Therapeutical Action of, 219
Samaritans, Ancient and Modern, 178
Sanitary Protection Association Report, 8
Sanitas, 332
Saturday Fund Statistics, 34
Sea Sickness, 312
Secret Remedies, 115
—— of Youth, The, 51
Sedox Absorbent Dressings, 28
Service of Meals in Asylums, The, 365
Sewage of Isolated Dwellings, The, 39
Sewing in Schools, 343
Sheffield Infirmary, 425
Siddons, Mrs. Chops, The Moral of, 23

Sincerity of Modern Medicine, The, 179
Skull, Formation of, 8
Smoke and Fog Nuisance, 22
Snake Bites, Remedy for, 278
Society Prevention Cruelty to Children, 312
Some American Hospitals, 158
Some Bloated Aristocrats of Physic, 243
Southport Infirmary, New, 360
Spasmodic Torticollis Treatment, 314
Special Hospitals, 255
Specialism and Individualism, 323
Speech of Monkeys, 408
Spinal Abscess, Discussion on Treatment of, 362
Spinal Caries, Laminectomy for Compression Paraplegia Due to, 42
St. Bede's Disinfectant, 268
St. John's Foundation School, 184, 248
St. Mary's Hospital, 8
Skulls, Excavation of, 262
Sleeplessness Complicating other Diseases, Treatment of, 346
Stands Scotland where it Did? 423
Student in Vienna, The 406
Suffering London, 129, 145, 157, 206
Suffering and Goodwill, Canon Newbolt on, 49
Sunderland Infirmary, 425
Sunlight Cure, The, 66
Sunspots and Insanity, 406
Sympathetic Ophthalmitis, 156
Street Collections, 290
—— Troughs and Glanders, 336
Syphons, Improvement in, 13
Teeth Deterioration, 406
Tennis or Golf for Ladies, 215
Thankless Unpopularity, 87
Throat Affection and Ear Ache, Cure for, 422
Tonsils, Hypertrophy of the, 38
Tobacco Smoke and Cholera, 358
Thyroid Extract from Myxœdema, 266 282
The State and the Shop, 353
—— Harmless Necessary Tart, 375
—— Latest Criticism, 306
—— Meaning of Pain, 309
—— Nine Circles, 188
—— Other Cheek, 211
Too Much Charity, 296
Town Children's Summer, 167
Toxteth Poor Law Infirmary, 248
Town Refuse, Destruction of, 422
Trachoma, Treatment of, 124
Treatment of Cough in Phthisis, 154
Trepanning and Epilepsy, 326
Trephining for Epilepsy, 331
Triticumina Wholemeal Bread, 268
Tumours of the Bladder, 379
Tumours on Spinal Chord, The Diagnosis of, 26

University College Hospital, Rebuilding of 8, 201
Unmarried Hampstead, Much Married Whitechapel, 19
Unqualified Assistants, 120

Vaccination, 102
—— In Italy, 406
Vegetarianism, Lady Paget on, 55
Vicarious Benevolence, 146
Victory, 210
Vitaline, 246
Vivisection, 194, 244

Wages versus Comfort, 84
Wanted—Locality, 130
Ward Walls, 32
Water as Stimulant, 102
Waterloo, Sir S., Resignation, 216
West End Hospital for Diseases of Nervous System, &c., 201
—— Ham Hospital, 104
—— London Hospital, 216
Westminster Hospital, 249
Who of Us is Fit to Practice? 215
Why Will Doctors not Save? 119
With Modern Eyes, 67
Women and Children's Hospital, Cork, 89
—— as Workers:—I. Where Women Most Fail, 389; II., Where Women Need Not Fail, 404
Working Men and Hospitals, 88
Worcester General Infirmary, 153
Young Women's Christian Association, 408

### THE SPECIAL NURSING SUPPLEMENT (MIRROR).

Abdominal Surgery, lxxxii

Aberdeen Nursing Association, cxlv

About Hospital Nures, clxxxi

Adelaide Hospital, Dublin, clxix

A Difficult Case, xlii

Advance Guardians, lxxiii

Allhallows, Ditchingham, xciii

American News, lxvii

Among the Institutions, xix, xxiv, xxxiv, xli, lvi

Another Lady Lecturer, clxxxi

Another's Reputation, cvii

Appointments: ii, viii, xviii, xxxiii, l, lxviii, lxxi, xc, cxi, cxxxii., cxl, cl, clvii, clxiii, clxxxv

A Private Asylum, iv.

—— Nurse's Report, clxxii

A Trained Nurse for Castor, lxxix

A Trained Nurse for Sidcup, clxxv
A Troublesome Case, lxii
Asylum Notes, lxxv, cv.
—— Attendants, xv, xc, lxv
A Warning, clvi
—— Note, lxxiii
—— to Nurses, lxxix
A Walking Tour, xxxiii

143512

Ayr County Hospital, cliii

Bathing at San Sebastian, clxxxi
Bedfordshire Nurses' Institute, lxxix
Behind Glass Doors, lxvii
Belsey v. McLeland, cxxi, clxxxiv
Bicester Nursing Home, i
Birmingham Institution, i
Blackpool Ladies' Sick Poor Association, lxi
Brabazon Home of Comfort, lxxxvii
Brassey Holiday Home, cliii
Brectin Victoria Nursing Association, cliii
Burton Institution, i
Bygone Uniforms, xix

Cambridge Home and Training School for Nurses, clxv
Care of the Feeble-minded, xxxvii
Cheltenham General Hospital, ci
Children, lxxxvi
Christmas Competitions, cvii
Cholera, clxiv
——— and Common Sense, clxxii
——— Lectures to Nurses, clxxii, clxxxv
——— Preparations at the Hospitals, clxxii
Clapham Maternity Hospital, lxxxvii
Clithero District Nursing Association, clxxxi
Cold Ash Hospital, lxxix
Cookery, xlv
Concert at London Hospital, lxi
Consumptive Patients, The Management of: I. The Infectiousness of Phthisis, cxxx; II. Disinfection of the Expectoration, cxxxviii; III. General Advice, cxlvi
Cork and Neighbourhood, lvi
Correspondence, xxxvii
Crieff Sick Nursing Association, cxxix
Crumpsall Infirmary, clxix

Danger of Developing into a Tyrant, The, lxvi
Death at Lambeth, cxxi
Derby Royal Nursing Institution, cliii.
Diet Dispensary, A, liii
District Nursing at Camberwell, clxxxi
District Nurses and Poor Law, cxlv

East End Mothers' Home, xiiix
Edinburgh Lady Medical Students, lxxix
——— Royal Infirmary, lxxix
Effects of Cleaning, cxxi
Endowed Bed, The, xxi
Essentials of Medical Electricity, cl
Examination of Nurses at Johns Hopkins Hospital, cxli
Experiences of Lowestoft Hospital, cxxxi
——— of a Nurse in Valparaiso, xlix, lvii

Facts and Foundlings, xli
Faddists of the Day, liii
Fever Hospitals, clxi
——— Nurses, clxi
Foresters and Salisbury Infirmary, clxxv
Four Months in a Hospital Ward, xxxv, xliii, li, lxxxiii, lxxxv, xcii, cvi

General Hospital, Birmingham, clxxii, clxxix
Graphic Summer Number, lxxxvii
Gravesend Nurses, cxxix
Greetings from Vancouver, xviii
Grosvenor Hospital for Women and Children, cv

Hamilton Association for Trained Nurses, xcviii, cv, cxxvii
He or She? xciii
Hermits of Dartmoor, clxxiv
Hints on Hands, cxv
Hints to Nurses, xviii, lxv, clxxiv
History of Registration, The, cxxvi
Holborn Guardians, ci
Holidays, lxxxvii, ci
Holiday Homes for Northern Nurses, clxxix, clxxxiv
Home Comforts for Epileptics, cxiii
——— for the Dying, A, xxi
Hospital Arrangements, clxxii
——— and Asylum Nurses, cxviii, clxxxv
——— Reports, lxi
House, The, xxi
How to Become a Medical Woman, clxxi, clxxvi
—— to Become a Dispenser, clxxxiii
—— to Procure Nurses for India Locally, clxiii

Instruction in Invalid Cooking for Training Schools, A Plan of, cix, cxvi
Invalid Children's Aid Society, ci
Insane of Montenegro, liii

Jersey, cv

Kensington District Nursing Association, cvii
Kitchen at Plaistow, clxi

Ladies' Conference at Sanitary Congress, clxxviii
Lady Doctors in India, iii
—— Lecturers, clxxv
—— Sanitarists, cxxxv
Lady Students, Edinburgh Royal Infirmary, cxlv
—— Students, clxix
Last Offices, lxvii
Lectures for Asylum Attendants. I.—Introduction, clv, clxii, clxx; II., Ventilation, clxxxii
——— to Nurses, lxi
Leicester Nursing Institution, lxvii
Lerwick, xxix
Lincoln Nurses, xxi
Little Children, i
——— Sisters of the Assumption, The, cli
London's End, clxxxiii
London Hospital Examination, cxiii
——— School of Medicine for Women, xxxviii
——— Temperance Hospital, liii
Long Life for Nurses, clxix
Longton Cottage Hospital, cxxvi, cxxxlv
Lowestoft Hospital, xxxvii, lvi

Male Probationers, xxxiii
—— Nurses, xiii, cxlii, clxv
Manners in the Midlands, cxxxiv
Mary Adelaide Nurses, cxxxvii
Massage, xviii, cxxxvii, clvi
Matrons, lviii
Matronship of Longton Cottage Hospital, cx, cxviii
Meath Home of Comfort, cliii
Medicine as Before, The, cxlii
Memorial to Nurse Coats, cvii
Metropolitan Fever Hospital, clxxii
——— and National Nursing Association, xlvii
——— Provident Medical Association,
Middlesex Hospital, lxxiii
Midwifery for Nurses, clxxxi
Midwives Bill, Proposed, lxiv, lxxvi
——— Registration of, xli
——— Registration, xxi, clxix
——— Proposed Bill for Registration, lxix
Milk and Scarlet Fever, clxxix

National Hospital, Queen's Square, lxvii
Naulahka, The, cxlix
New Home for Invalids, Cannes, clxi
News from South Africa, vii
Nightingale Fund, cxlvii
Northern Workhouse Nursing Association, liii
Notes from Australia, x, xlvi, xlviii, cxvii
——— on Novelties, xc, cxliii
Notice to Nurses, clxxxv
Nottingham and Notts Nursing Association, lxi
Nourishment for the Needy, clxxxi
Nurses and Basuto War Medal, cxviii
——— Beneficial Association, i
——— Bookshelf, xxv, cxxiv
——— at Colney Hatch, xlv
——— Co-operation, vii, xviii, xxxiii, liii, lxxix, cxiii, clxix
——— For the English in India, xxv, xxxii
——— For India, xlv., ci
——— How to Procure, civ
——— and King's College Hospital, cv
——— Journal Club, The, cxxv
——— Outfit, lxxxii
——— Recreation, clxxv
——— Residential Club, lxvi
——— Wages, lxxii
Nursing in Asylums, cxxvi
——— at Carlisle Infirmary, cxxxlv
——— of Children, viii, xvi
——— Homes, xxxiv, xl, l, lviii, lxxxi, xci, cx
——— Birmingham General Hospital, clv
——— and Paying Patients, vii
——— and Hygiene, lxxvii
——— of the Insane, ii, xxxi, xxxix
——— Institution at Birmingham, liii
——— Outdoor Paupers, cxxix
——— At the Royal, xxix
——— of the Sick English Soldier in India, lxxxix
——— Small Pox Cases, lxxvii, xc
——— Uniforms, xi, xxxiii
——— in Workhouse Sick Wards, lv
——— Workhouse Infimary, lxx

Observant Womankind, xxix
Obstetric Hints for the Use of Midwives, lxxi
Old and New, The, xvii
On Board Antwerp Boat, xix
— Commission, cvii
— The Nursing of Children, viii
One Who Wants to Know, cxviii
Our American Sisters, cxxiv, cxxxiii
—— Australian Letter, clxxviii
—— Indian Letter, cxxxix, clxxviii

Personal Inspection, lxvii
Pharisee, Mrs., v
Pleasing Comparison, xxix
Poisons as Infantile Nourishment, lxxxvii
Poor and Friendless, lxi
Practical Points, clxi
Preston Royal Infirmary, cliii
Prince Alfred Hospital, Sydney, clxxv
Private Nurse, The, i
——— Nurses, clxv
——— in Dublin, xciii
——— and Their Ways, lxxi, lxxvii, lxxxvi, lxxxiii
Prizes, School of Medicine for Women, ci
Probationers, cxxiii
——— "Time off Duty," xxi
Progress of District Nursing, xxi
Prospects of Southampton Nurses, clxi
——— of Private Nurses, lxiii
Queen Charlotte's Lying-In Hospital, lxxiii
Queen Victoria's Jubilee Institute, iv, cv, cxxix
——— in Dublin, lxxxvii
——— in Scotland, ci

Radcliffe Infirmary, Oxford, vii
Random Evidences, lxi
Reading to the Sick, iii, ix, xv, xxiii, xxxi, xxxix, xlvii, lv, lxiii, lxix, lxxv, lxxxi, xci, xcvii, cxi, cxix, cxxv, cxxxiii, cxliii, cxlvii, clvi, clxiii, clxxii, clxxxv
Redruth, lxxiii
Register of Trained Nurses, lxxxiv
Registration of Nurses, The, i, vii, xiii, cxviii, cxxi
——— Lords' Report, xcv
——— Midwives, The, xciii
Relieve the Surplus Nursing Energy, xxiv
Removal of the Brassey Home, clxxii
Result of a Chill, clxxiv, clxxx, clxxxvi
Ring-worm and Mob-caps, xcix
Royal Cornwall Infirmary, clv
——— Berks Hospital, Reading, cli
——— British Nurses' Association, xxix, cxxi
——— Free Hospital. clxxxi
——— National Pension Fund for Nurses, clx x i clxxxv
——— South Hants Infirmary, xciii
Rural Nursing and Jubilee Institute, lv

Samaritan Society at St. Thomas's, xxxvii
Seasonable Suggestion, A, clxix
Select Committee on Metropolitan Hospital, ciii
Significant, ix
Sisters, cx
Small-Pox and Infectious Fever Nursing, xcix
Society Employment of Women, cvii
Some Australian Experiences, lxxxiv, clvii, clxvii
Staff Nurses, cxv
Stalybridge Sick Nursing and Mothers' Help Society, ci
Sterilised Salt Solution, lxxxii
St. George's Hospital Nurses, xlv
St. Kilda, xciii
St. Mary's Home, Southampton, i
St. Olave's District Nursing Association, cxxix
——— Infirmary, lxxvii
St. Patrick's Home, clxxxi
Stockholm Nurses, xiii
Stockton District Nursing Association, xlv
——— and Thornaby District Nurses, xciii
Sutton, Dr., and His Students, liii
Sunderland Institute,
Supplement,
Switzerland, A Holiday in, l, xc, cxviii
Sydney Hospital, cvii

Talking Shop, xviii, xxvi
——— about Food, vi; Drink, vi
Tall or Short, clxix
Tea and Tea Drinking, lxxix
—— and Talk, cxiii, lxxiii
The Co-operative System, xix
The Real Mrs. Skeffington, cxxviii, cxxxv
Three Years' Training, cxiii
Toronto General Hospital Training School, xcvi
Trained Male Nurses, cxxvi
Trains and Trained Nurses. lxxix
*Truth* and the London, cxiii
Twenty Years Ago, xiii
Uniforms, cxlii
Uniform, cxiii

Ventilation, Disinfection, and Diet, xiv, xxii, xxx, xxxviii, xlvi, liv, lxii, lxiv, lxviii, lxxx, lxxxviii, xciv, cii, cviii, cxiv, cxxii
Victoria Hospital, Hull, cxxxvii
Victoria Hospital, Children, clxxv
Visitors in General Hospital Wards, clxvi

Wardmaids, cxxxi
Warwick Nursing Association, cxiii
Well Done, Aberdeen, cxxix
Welsh Branch of the Q.V.J.I.N., xlv
Western-Super-Mare, cxxix
Westminster Hospital Samaritan Fund, lxvii
Why Probationers Break Down, cxlviii
Women and Fire Drill, clxix
Woolwich District Nursing, xxxvii
Worcester General Infirmary, lxxiii
Work and Health, lxxiii
Workhouse Infirmary Nursing Association, lxi
Would-be Suicides, xxxiii
Wrentham College Training Home, lxxiii

Zenana Bible, xl

Printed by W. H. and L. Collingridge, "City Press," 148 and 149, Aldersgate Street, London, E.C.

**Notices of Births, Deaths, and Marriages,** are inserted in this column at a charge of 2s. 6d. for an announcement not exceeding four lines.

### NOTICE TO CORRESPONDENTS.

All MS., Letters, Books for Review, and other matters intended for the Editor should be addressed The Editor, The Lodge, Porchester Square, London, W.

The Editor cannot undertake to return rejected M.S., even when accompanied by stamped directed envelope.

**Notice to Advertisers and Subscribers.**

All Advertisements, Orders for Copies of The Hospital, and Business Communications should be addressed to The Publisher, *not to the Editor*, The Hospital, 140, Strand, W.C.

The Cost of Subscription, post free, is as follows:—Per week, 2½d.; per quarter, 2s. 6d.; per half-year, 5s.; per annum, 8s. 6d. Foreign:—Per annum, 10s. 8d.; payable, in all cases, in advance.

**"Burdett's Hospital Annual," 1891—1892.**

Third year of publication. 600 pp. Boards, gilt lettering. A most complete guide to British and Colonial Hospitals, Dispensaries, Nursing and Convalescent Institutions, and Asylums. Price 3s. 6d. Published by The Scientific Press (Limited), 140, Strand, W.C.

**NOTICE.—The Index for Vol. X. (ending September, 1891), is on sale at the Office of this Journal, price 2½d., post free.**

### Scraps and Gleanings.

The Queen has graciously sent a donation of £20 to the Irish Distressed Ladies' Fund.

The sum of about £6,000 was realised during the first two days' sale of the Greenock Infirmary Bazaar.

The Committee of the Leeds Royal Infirmary appeal for £15,000 to cover the total cost of the new wing now being added to the hospital.

One of the April magazines has a paper on "The Harvest of the Sea." It is—very appropriately—the work of a writer named Salmon.

The Victoria Hospital, Folkestone, has received £100 from Miss Ogilvie as a thankoffering for having been spared from the recent scourge.

Mrs. Rimmer, Lord Salisbury's old nurse, died last week at Chidwell, near Liverpool, aged 80. It is reported that whenever the Premier was in that part of the country he never failed to pay her a visit.

It is a matter for regret that the Dublin Hospital Sunday Fund for the past year amounted only to £3,720. On comparison with the average of the first three years, this sum shows a falling off of about £200.

A second concert in aid of the building fund of the new wing of the North London Hospital for Consumption is to take place on Wednesday, April 6th, at St. James's Hall, when Handel's "Samson" will be performed.

Dr. George Buchanan has expressed his intention of resigning the post of Medical Officer to the Local Government Board at the close of the present financial year, and his resignation will take effect from the 31st inst.

The Chief Secretary for Ireland has contributed £25 towards the Memorial Fund to the late Duke of Clarence, which is being raised by the Leeds District Nursing Association, for opening and maintaining homes for nurses.

His Royal Highness the Duke of Cambridge, President of the German Hospital, Dalston, has kindly promised to take the chair at the forty-seventh anniversary dinner, at the Whitehall Rooms, Hotel Metropole, on Wednesday, May 11.

A lad named Alfred Reader recently met with an almost miraculous escape. He fell a distance of 50 feet down an hydraulic lift shaft upon an iron flooring, and when picked up was found to be suffering only from a slight cut on the head, and a few bruises about the body.

At the annual meeting of the Hull Royal Infirmary it was proposed that the two Hull Sailors' Homes and other institutions for the relief of human suffering in that town, should combine with the infirmary in the effort to establish a convalescent home at Withernsea.

Dr. Wynn Westcott has been elected by the vestry of St. Mary, Islington, to succeed the late Dr. Meymott Tidy as Medical Officer of Health, *pro tem.*, for the parish. The permanent appointment cannot be made for some months, owing to the provisions of the new Public Health Act.

Sir Whittaker Ellis, M.P., has withdrawn his consent to be nominated as a candidate for the treasurership of the Foundling Hospital, the general opinion of the Governors being in favour of the election of Mr. Robert Grey, who has been for many years a member of the governing body.

At the annual meeting of the Royal National Hospital for Consumption, Ventnor, it was announced that Her Majesty the Queen has become an annual subscriber of £10. During the year 722 in-patients were treated. The receipts amounted to £10,103 18s. 11d., and the expenditure to £10,941 7s.

The financial statement of the Rhyl Royal Alexandra Children's Hospital showed that the total receipts for the past year amounted to £3,899. The payments by patients amounted to £1,777; workers and pupils, £573; and for nurses' services, £277. The expenditure nearly balanced the receipts.

The Lord Chancellor announced at the fortieth annual dinner of the Hospital for Sick Children, Great Ormond Street, that an anonymous donor had given £500 to the hospital. Ten thousand pounds is needed to pay for the completion of the institution, so that the new wards may be opened free of debt. The subscriptions reached the total of £4,300.

Mr. Egerton Burnett has most generously promised to erect a new cottage hospital at Wellington, Somerset. The founder and donor has applied to the Princess of Wales, asking permission to name the men's ward in memory of the late Duke of Clarence. Her Royal Highness has graciously consented, and wishes the name to be "The Albert Victor Christain Edward Ward."

At the annual meeting of the Bristol Royal Infirmary the Committee stated with regret that the debt due to the Treasurer increases yearly, and now amounts to nearly £3,700. So useful a charity ought surely to find more than 1,100 firms and individuals willing to support it, and the sum of £2,700 contributed in this way is a very inadequate representation of the wealth of this great city. The whole cost of the working of the institution for the year has been £11,788.

The fifty-third report of the annual court of the Royal Berkshire Hospital states 1,145 in-patient, and 1,687 out-patients were admitted during the year. The Treasurer's report shows that, by the large sum of £2,238 14s. 9d. received in donations, he has been enabled to clear off the adverse balance of £437 13s. 2d., with which the year commenced, and to carry over a balance of £139 17s. 8d. for the current year. Improvements in the servants and nurses' wing have also been made to the sum of £1,000

The annual report of the Leeds Infirmary for 1891 states that 5,125 in-patients were admitted, and the number remaining in the hospital at the end of the year was 306, making a total of 5,431. 28,864 out-patients were treated, against 26,585 in the previous year. The average daily number of beds occupied was 39; the cost of each in-patient, £2 15s. 4½d., and of each out-patient, 1s. 3½d. The treasurer's receipts were £20,154 12s. 6d., of which the two principal items were, £5,715 12s. 3d. (dividends, interest, and rent), and £5,114 2s. 5d. (workpeople's contributions).

### NOTICES.

### FURNITURE AND APPLIANCES.

We purpose giving a descriptive account with illustrations of all the new appliances and furniture used in hospitals and asylums, and we desire to secure the co-operation and the superintendence of the medical staff, secretaries, and matrons, so that the whole ground may be covered by this section, which should prove very interesting and helpful in many ways. Each institution which has any fittings or furniture which are considered specially suitable or novel are invited to send a description with sketch for publication in this column. All communications should be marked "Furniture and Appliances," and addressed to the Editor of The Hospital, 140, Strand, W.C.

### PRACTICAL POINTS.

Questions are invited for this column on any point in the administration of hospitals and asylums which may prove of difficulty or interest to our readers. We propose to give a few typical questions and answers, and shall continue them from time to time providing the idea is taken up by those for whose benefit it is chiefly intended. All communications should be marked "Practical Points," addressed to the Editor of The Hospital, 140, Strand, W.C.

### THE STUDENT OF MEDICINE.

[All communications for this Supplement should be addressed to The Editor of The Hospital, at 140, Strand, with the words, "Students' Column," written in the left hand top corner of the envelope.]

### APPOINTMENTS.

G. H. A. C. Berkeley, M.R.C.S., L.R.C.P., appointed House Physician at the Middlesex Hospital. H. B. Bolus, M.R.C.S., L.R.C.P., appointed Assistant House-Physician to Guy's Hospital. T. B. P. Davies, M.B., B.S.Lond., appointed House-Surgeon to Guy's Hospital. H. E. Durham, M.B., B.C.Cantab., appointed Assistant House-Surgeon to Guy's Hospital. W. H. Fisher, M.B., B.C.Cantab., appointed Assistant House-Physician to Guy's Hospital. A. L. Macleod, M.B., C.M.Univ.Glasg., appointed Junior House-Surgeon to the Macclesfield General Infirmary. Sidney H. C. Martin, B.Sc., M.D., F.R.C.P.Lond., M.R.C.S.Eng., appointed Assistant Physician to the Brompton Hospital for Consumption. H. N. D. Milligan, M.B., C.M.Edin., appointed Junior House-Surgeon to the Bradford Infirmary. W. H. Murdoch, M.D., Senior Resident Medical Officer, Parish Infirmary, Liverpool, appointed Physician to Convalescent Home for Children, Fulwood, and Surgeon to Dinglemount Home for Epileptics. A. T. Rake, M.B., B.S.Lond., appointed Assistant House-Surgeon to Guy's Hospital. Albert Max Sully, L.R.C.P.Lond., M.R.C.S.Eng., appointed Resident Medical and Surgical Officer to the Jaffray Suburban Hospital, Birmingham. Theophilus William Trend, M.D., St.And., M.R.C.S.Edin., appointed Physician to the Royal South Hants Infirmary, Southampton. F. M. Turner, M.B., B.C.Cantab., appointed House-Physician to Guy's Hospital. F. S. Wood, M.R.C.S., L.R.C.P., appointed House-Surgeon to Guy's Hospital. J. Young, M.B., B.S.Lond., appointed House-Physician to Guy's Hospital. Charles C. Heywood, M.A.M.B.Cantab., M.R.C.S.Eng., appointed House-Surgeon to the Salford Royal Hospital. Gervasse E. Newby, M.R.C.S.Eng., L.R.C.P.Lond., appointed Junior House Surgeon to the Salford Royal Hospital.

### VACANCIES.

### Resident Medical Officers.

The following vacancies are announced this week, the amount of salary in each case being given after the name of the institution:

Buckingham General Infirmary, Aylesbury, Surgeon and Apothecary, doubly qualified—Salary £80 per annum, rising £10 yearly to £100, with Board, &c.

Derbyshire Royal Infirmary, Assistant House-Surgeon—Salary £10 for first six months, £25 for second six months, with Board, &c.

Hulme Dispensary, Manchester, House-Surgeon, doubly qualified—Salary £140 per annum, &c.

Londonderry Lunatic Asylum, Assistant Medical Officer—Salary £100 per annum, &c.

Manchester Southern and Maternity Hospital, House-Surgeon—Honorarium at the rate of £75 per annum.

Royal Free Hospital, Gray's Inn Road, Senior Medical Officer, doubly qualified—Salary £100 per annum, with Board, &c.

Royal Free Hospital, Gray's Inn Road, Junior Medical Officer—Board, &c.

Royal Pimlico Dispensary, 104, Buckingham Palace Road, S.W., Medical Officer and Assistant Secretary—Salary £100 per annum, &c.

### Non-Resident Medical Officers.

Charing Cross Hospital, Pathologist and Curator—Salary £100 per annum.

College of State Medicine, Research Scholarship, tenable for one year—Salary £100.

Manchester Southern and Maternity Hospital. House-Surgeon.

South Devon and East Cornwall Hospital, Plymouth, Honorary Assistant House-Surgeon.

Victoria Hospital for Children, Queen's Road, Chelsea, Honorary Medical Officer to New Convalescent Branch at Broadstairs to be opened in May.

### NOTES FROM THE SCHOOLS.

Guy's Hospital.—The present issue of the "Gazette" contains the notes of a clinical lecture by Dr. Hall White on "Cardiac Pain and Distress."

St. Thomas's Hospital.—The "Gazette" for March contains an article by Dr. H. W. G. Mackenzie, conveying some excellent suggestions on the subject of Notetaking.

### PASS LISTS.

### Society of Apothecaries of London, March, 1892.

The following candidates passed:—

In Surgery.—A. D. Bensusan and W. T. B. Donelly, King's College Hospital; H. J. Forster, Westminster Hospital; R. T. Gilmour, St. Mary's Hospital; J. D. Hessey, Middlesex Hospital; F. V. H. Mossman, Owens College, Manchester; N. F. Roth, St. Mary's Hospital; S. A. L. Sodipo, Newcastle-on-Tyne; E. J. Steegmann, St. Mary's Hospital; R. S. Whitford, Charing Cross Hospital; J. Wood, St. Thomas's Hospital.

### The Hospital Advertiser.

### THE STUDENT OF MEDICINE—(*continued*).

In Medicine, Forensic Medicine, and Midwifery.—P. Goold, Cork; G. B. Hillman, Yorkshire College, Leeds; F. V. H. Mossman, Owens College, Manchester.

In Medicine and Forensic Medicine.—W. D. Akers, St. Mary's Hospital.

In Medicine and Midwifery.—V. H. Barr, Guy's Hospital; J. Kyffin, London Hospital; W. K. Steele, Guy's Hospital.

In Medicine.—C. A. Davies, Owens College, Manchester; E. E. Frazer, Guy's Hospital; G. Grace, Bristol.

In Forensic Medicine and Midwifery.—C. O'Sullivan, London Hospital; W. A. Ward, Middlesex Hospital.

In Midwifery.—J. Dulberg, Owens College, Manchester; H. F. Ealand, St. Mary's Hospital; A. C. Fenn, St. Bartholomew's; W. Fowler, Durham University; W. M. Palmer, Charing Cross Hospital; R. A. Smith, Edinburgh.

To Messrs. Barr, Bensusan, Donelly, Dulberg, Forster, Goold, Hessey, Mossman, O'Sullivan, Palmer, Steegmann, and Ward was granted the diploma of the Society entitling them to practise medicine, surgery, and midwifery.

### Royal College of Physicians, England.

The following gentlemen, having conformed to the bye-laws and regulations and passed the required examinations, were, at a meeting of the College on January 28th, admitted Licentiates:—

Adams, A., St. Bartholomew's
Adams, E. W., King's College
Adams, W. F., King's College
*Archer, S. L., St. Bartholomew's
Armstead, H. W., St. Bartholomew's
Attlee, J., St. Bartholomew's
Barker, C., St. Bartholomew's and Sheffield
Bell, J. A., King's College
Biddle, H. G., Guy's
Billson, C., St. Thomas's
Bishop, G. T., Charing Cross
Blackwell, A. S., St. Bartholomew's
Bolus, H. B., Guy's
Braithwaite, C. B., Guy's
Browne, J. J., Guy's
Brownlow, H. L., St. Bartholomew's
Burden, H., St. Thomas's
Cooke, W. H., St. George's
Cooper, A. T., University College
Day, J. H., Charing Cross
*Dolamore, W. H., St. Mary's
Dorman, M. R. P., St. Thomas's
Drake, W. E., St. Thomas's
*Eccles, R. B., St. Thomas's
Ellis, F., London
English, T. H., London
Evans, J., Liverpool
Everett, E. W., St. Bartholomew's
Fearnley, J., Leeds
Fennings, A. A., St. Mary's and Durham
Finley, H., University College
*Fraser, R. C., Guy's
Freeland, D. L., Middlesex
Glover, L. G., St. Bartholomew's
Graham, C. H., St. Bartholomew's
Green, J., Manchester
*Greenhalgh, A., Manchester
Griffiths, G. B., St. Bartholomew's
Haslam, W. A., Guy's
Haydon, A. G., St. Bartholomew's
Hayes, W. A., University College
Higgs, W. A., Guy's
Hignett, L. W., Edinburgh
Hopkin, R., London
Hutton, J. W., Leeds
Iliewicz, E. M., St. Mary's
Jerome, G. P., Birmingham
Jones, L. G. D., Manchester
Jones, W. B., St. Bartholomew's
Kendall, H. W., Middlesex
Kitching, C. A., London
Lacey, A. R., Guy's
Lee, S. H., Middlesex
*Leicester, T., St. Thomas's
Lemarchand, A. W., St. Bartholomew's
Lewarne, F., St. Bartholomew's
Lewis, H. W., Westminster
Lister, S. R., St. Bartholomew's
Lloyd, F. G., St Bartholomew's
Lord, P., Guy's
Lumley, F. D., Guy's
Lunn, C. R., Birmingham
*McAnally, A. A., King's College
McKinnon, A. A., King's College
McMichael, A. W., Birmingham
Marin, F. B., Westminster
*Martland, T., Manchester
Matthews, W. R., London
Miller, J., St. Bartholomew's
Minshall, A. G., University College
Morgan, H. de R., St. George's
Morrison, J., St. Bartholomew's
*Nash, C., King's College
Newby, G. E., University College
Nicholls, S. R., London
Norman, G., Westminster
Norris, A. E., Guy's
Nuttall, W. W., St. Bartholomew's
Passmore, J. E. S., London
Pauling, W. T., St. Thomas's
Phillips, P. C., St. Thomas's
Pickford, J. S., Manchester
Pitt, T., Bristol
Porter, F. C., Charing Cross
Powrie, P. C., University College
Prall, C. B., St. Thomas's
*Prosser-Evans; J., University College
Prynne, H. V., Middlesex
Ralph, C. H. D., Guy's
Ramsay, G., King's College
*Richardson, W. J., King's College
Roth, W. E., St. Thomas's
Rowell, J. G., Leeds
Rutter, F. B., London
Sargent, H. C., London
Senior, H. D., Charing Cross
Star, P. H. M., Bristol
Steegmann, E. J., St Mary's
Thomas, H., St. Bartholomew's
Thomas, W., London
Thorne, B. B. T., St. Bartholomew's
Turner, J. G., St Thomas's
Turner, O. P., University College
Visick, C. H. C., University College
Voisin, E. O. B., University College
Wallace, F. G., St. Thomas's
Warner, T., King's College
Waters, H. G. St. Thomas's
Watkins, D. J. G., St. Bartholomew's
Weichert, C. J., University College
Whiteford, C. H., St. Bartholomew's
Whitehead, A. L., Leeds
Wiglesworth, T. R., Bristol
Wild, H. S., St. George's
Wilkinson, H. B., Guy's
Williamson, J. H., Manchester
Wood, C. G. R., Bristol
Worthington, H. E., Guy's
Wrench, E. B., Cambridge and St. Thomas's
Wylie, J. T. R., St. George's

* Candidates who have not presented themselves under the regulations of the Examining Board.

### ATHLETICS.—Football.

Association Final Cup Tie.

St. Bartholomew's Hospital v. St. Mary's Hospital.—This tie was re-played at Leyton on Wednesday, March 24th, and resulted in a win for St. Bartholomew's by two goals to one. The play on both sides was somewhat indifferent.

Resident Medical Superintendent.

